# Improved access to and continuity of primary care after attachment to a family physician: longitudinal cohort study on centralized waiting lists for unattached patients in Quebec, Canada

**DOI:** 10.1186/s12875-022-01850-4

**Published:** 2022-09-16

**Authors:** Mélanie Ann Smithman, Jeannie Haggerty, Isabelle Gaboury, Mylaine Breton

**Affiliations:** 1 Université de Sherbrooke, 150 place Charles-Le Moyne, bureau 200, Longueuil, Québec, J4K 0A8 Canada; 2 St. Mary’s Research Center, McGill University, 3830 avenue Lacombe, Hayes Pavilion, suite 4720, Montreal, Québec, H3t 1M5 Canada

**Keywords:** Primary health care, Attachment, Patient rostering, Observational study, Health services accessibility, Family physicians, Physicians, primary care, Waiting lists, Continuity of patient care, Longitudinal studies

## Abstract

**Background:**

Having a regular family physician is associated with many benefits. Formal attachment – an administrative patient-family physician agreement – is a popular feature in primary care, intended to improve access to and continuity of care with a family physician. However, little evidence exists about its effectiveness. In Quebec, Canada, where over 20% of the population is unattached, centralized waiting lists help attach patients. This provides a unique opportunity to observe the influence of attachment in previously unattached patients. The aim was to evaluate changes in access to and continuity of primary care associated with attachment to a family physician through Quebec’s centralized waiting lists for unattached patients.

**Methods:**

We conducted an observational longitudinal population cohort study, using medical services billing data from public health insurance in the province of Québec, Canada. We included patients attached through centralized waiting lists for unattached patients between 2012 and 2014 (*n* = 410,140). Our study was informed by Aday and Andersen’s framework for the study of access to health services. We compared outcomes during four 12-month periods: two periods before and two periods after attachment, with T0–2 years as the reference period. Outcome measures were number of primary care visits and Bice-Boxerman Concentration of Care Index at the physician and practice level (for patients with ≥2 visits in a given period). We included age, sex, region remoteness, medical vulnerability, and Charlson Comorbidity Index as covariates in regression models fitted with generalized estimating equations.

**Results:**

The number of primary care visits increased by 103% in the first post attachment year and 29% in the second year (*p* < 0.001). The odds of having all primary care visits concentrated with a single physician increased by 53% in the first year and 22% (*p* < 0.001) in the second year after attachment. At the practice level, the odds of perfect concentration of care increased by 19% (*p* < 0.001) and 15% (*p* < 0.001) respectively, in first and second year after attachment.

**Conclusion:**

Our results show an increase in patients’ number of primary care visits and concentration of care at the family physician and practice level after attachment to a family physician. This suggests that attachment may help improve access to and continuity of primary care.

**Supplementary Information:**

The online version contains supplementary material available at 10.1186/s12875-022-01850-4.

## Background

Patients with a regular primary care provider – most commonly a family physician – benefit from better preventive care, disease management, care coordination, access to care, continuity of care, and health outcomes [1–6]. Patients who have a regular provider use more primary care services, suggesting better access to primary care than patients without a family physician [7–10]. In addition, increased continuity of care, particularly with a family physician, is associated with decreased hospitalizations, fewer emergency visits, more preventive care, improved patient satisfaction, safer prescribing, lower rates of major adverse events and reduced mortality [11–17]. Conversely, patients without a regular primary care provider often experience access barriers, lower continuity of care, less comprehensive care, a lack of care coordination, more unmet needs, and often rely on walk-in clinics and emergency departments, where care can be suboptimal, particularly for patients with complex needs [2, 7, 18–21]. Previous studies compare patients with and without a regular family physician, but these groups have different characteristics that may influence and bias results [5, 7, 10, 19, 22–26].

In countries such as France, Germany, the Netherlands and Norway, less than 10% of the population reports not having a regular provider [27]. In contrast, across Canada that proportion is 17.1%, ranging from 13.7% in the province of Ontario to 21.1% in Quebec [27].

Formal attachment – also known as rostering, empanelment, enrollment or patient registration – is a policy intended to improve access to and continuity of care with a family physician. Attachment involves an administrative patient-provider agreement and is meant to assure access to a regular primary care provider or team who is most responsible for a patient’s care [28, 29]. Internationally, attachment is increasingly widespread as a building block of primary care [30, 31]. It is a key feature of the patient medical home, and supports practice management (e.g., establishing panel size, balancing supply and demand) and health system planning (e.g., accountability, performance, provider remuneration, resource allocation) [29–36]. However, very little research has evaluated the effects of attachment. Several studies have examined specific primary care models that attach patients, making it difficult to disentangle the effect attributable to attachment from other features of these delivery models (e.g. team-based care, after-hours care) [37–41]. Research suggests that, at a populational level, implementation of attachment policies may have limited impact on indicators of having a regular provider and continuity of care [42].

Quebec has, since 2009, implemented formal attachment across all primary care models, however many patients remain unattached because they cannot find a family physician accepting new patients. The priority placed on delivering services to their attached patients also makes family physicians less available to see unattached patients.

To help increase attachment, Quebec implemented centralized waiting lists for unattached patients – the *guichets d’accès pour la clientèle orpheline*. As of 2022, over 900,000 patients were waiting for attachment on these lists. Other Canadian provinces also have similar centralized waiting lists [43]. Previous research suggests that centralized waiting lists are effective to increase attachment [44]. However, a number of challenges remain, including large regional variations [45], long wait times (over 500 days on average), physicians’ preference for attaching healthy patients [44, 46], and inequities in attachment for patients from disadvantaged areas or with complex needs [44, 47–49]. Finally, how attachment through centralized waiting lists influences access to and continuity of primary care has not been evaluated.

Quebec’s centralized waiting lists for unattached patients create a unique opportunity to observe how attachment influences access to and continuity of primary care in previously unattached patients. Evaluating attachment is relevant to inform primary care policy, planning and delivery. The **objective** of this study was to evaluate changes in access to and continuity of primary care associated with attachment to a family physician through Quebec’s centralized waiting lists for unattached patients. Specifically, we hypothesized that after attachment through the centralized waiting list, the number of primary care visits would increase and be more concentrated with one family physician and primary care practice, compared to before attachment.

## Methods

### Study setting

Quebec is the second largest province in Canada with a population of about 8.5 million. The provincial tax-based healthcare system provides universal health insurance coverage for medical services. The main organizational model for the delivery of primary care services is the Family Medicine Group (*Groupe de médecine de famille)*: 62% of the population is attached to a family physician practicing in one of 340 such clinics [50]. Family Medicine Groups are composed of six or more family physicians working together in collaboration with an interdisciplinary team of allied health professionals (e.g., nurses, social workers, community pharmacists). Other models of primary care, including solo practices, groups practices, local community health centers, exist alongside Family Medicine Groups.

In Quebec, family physicians practicing across all models of primary care are strongly encouraged to formally attach patients [28]. Family physicians often prioritize delivering care to their attached patients. Although attached patients are free to visit providers other than their family physician, access to other providers remains limited with the notable exception of providers practicing within the same Family Medicine Group. Because of the focus on delivering care to their attached patients, family physicians have fewer availabilities for other patients. As a result, unattached patients have limited options to access primary care, often relying on walk-in clinics and emergency departments.

Centralized waiting lists are meant to help unattached patients become attached to a family physician. Registration on the centralized waiting list can be completed on a voluntary basis by the patients themselves or by another person on their behalf (e.g. by a social worker). Patients are then prioritized based on medical needs into five categories from most urgent to least urgent, based on self-reported health information and, if needed, telephone evaluation by a nurseif needed. Available family physicians then make a request for new patients, generally indicating the number of patients they are willing to attach and sometimes preferences for types of patients [48]. A nurse or administrative clerk then assign patients to the family physician based geographic location, needs-based priority, time since registration on the list and physician preferences. The physician’s practice then contacts the patient to book the first appointment. Physicians can refuse an assigned patient and return them to the centralized waiting list – for instance, if the patient is unreachable, lives too far from the practice or has a condition that is incompatible with the physician’s scope of practice. Patients may also refuse assignment to a family physician. Refused assignments are managed locally and informally by the centralized waiting list staff and medical coordinator (a family physician tasked with liaising between the centralized waiting list and local family physicians). Attachment is formalized through an administrative agreement signed upon the patient’s first visit to their new family physician. This agreement is linked to family physician fee codes, and physicians receive a one-time premium to attach a new patient, ranging from CAD $19 - $300 depending on the patient’s medical needs.

### Conceptual framework

The two outcomes of interest in this study were access to and continuity of primary care. Our conceptualization of access was informed by Aday and Andersen's framework for the study of access to medical care [51–53] in which “realized” access is viewed as the actual and observable utilization of services, resulting from successful entry into the health system. The framework is widely used for research on access, and identifies individual determinants of health care utilization: predisposing, enabling and need determinants [51–53]. The framework also identifies continuity as an outcome of access and suggests that fragmented and dispersed care may reflect a lack of appropriate access to care [53].

The notion of continuity of care is used in a variety of ways, with three types of continuity appearing in the medical literature: relational, informational and management [54]. While the three are, to some extent, intertwined, relational continuity is emphasized in primary care, where the provider takes principal responsibility for a patient over time and across multiple episodes of care. In primary care research, relational continuity is often inferred from the extent to which a patient concentrates their care in the same physician or primary care practice. Concentration of care often serves as a proxy for the trust and understanding that characterize the therapeutic relationship. Although all three types of continuity are important to achieving high quality care, relational continuity – and concentration of care as a proxy – was emphasized in this study because it is an explicit aim of centralized waiting lists and attachment to improve relational continuity.

Given these definitions, we hypothesized that attachment to a family physician, as an enabling determinant, would increase “realized” access (utilization) and relational continuity (concentration) of primary care.

### Study design and population

We conducted an observational longitudinal population cohort study [55] of patients attached to a family physician through centralized waiting lists for unattached patients in Quebec between January 1st 2010 and August 31st 2015. We used medical services billing data to compare patients’ utilization of primary care pre- and post-attachment. This study is reported in accordance with the Strengthening the Reporting of Observational Studies in Epidemiology (STROBE) guidelines [56] (see Appendix 1 for checklist).

### Data source

We used health administrative data from the *Régie de l’Assurance Maladie du Québec* (RAMQ) – Quebec’s single-payer insurance board. Family physicians’ remuneration is mainly fee-for-service, supplemented with some other forms of remuneration (e.g., incentives, salary, mixed remuneration). As elsewhere in Canada, fee-for-service accounts for approximately 70% of the average gross clinical payment to family physicians [57, 58]. This allows most medical visits to be tracked through RAMQ billing data and confers good validity to measures of medical services utilization.

### Participant selection

The cohort included all patients who had a billing code for attachment to a family physician through Quebec’s centralized waiting lists between January 1st, 2012 and August 31st, 2014 (RAMQ fee codes: 19951, 19,952, 19,956). These fee codes are only used for centralized waiting list attachments. We excluded patients who had multiple attachment fee codes within the study period, signalling multiple pre/post attachment periods. We also removed patients who were less than 1 year old at the time of attachment, as they lacked sufficient pre-attachment data.

### Outcome variables

The study variables are detailed in Table 1.

**Table 1:** Summary of variables

Variables	Description	Aday and Andersen’s Framework for the study of access (53)
Number of visits to family physicians	Number of visits to family physicians in a 12-month period.	Measure of realized access and utilization of primary care
	Discrete visits counted as ≥1 billing code, per date, family physician, and primary care location	
	Continuous variable	
Concentration of Care Index – family physician level	$COCI(FP)=\frac{\sum_{i=1}^k{n}_i^2-N}{N\left(N-1\right)}$ k = total number of family physicians visited *N* = total number of visits during the year n_i_ = number of visits to physician i	A proxy for relational continuity of care
	Different family physicians were identified using RAMQ unique physician identifier, physician speciality and type of organization to select primary care locations.	
	Continuous variable, ranges from 0 (all visits to different providers) to 1 (all visits to the same provider)	
Concentration of Care Index – practice level	$COCI(P)=\frac{\sum_{i=1}^k{n}_i^2-N}{N\left(N-1\right)}$ k = total number of practices visited *N* = total number of visits during the year n_i_ = number of visits to practice i	A proxy for continuity of care with the practice
	Different practices were identified using RAMQ unique identifier for delivery organization, unique physician identifier, physician speciality and type of organization to select primary care locations. Non-physician visits are not available in RAMQ data.	
	Continuous variable, ranges from 0 (all visits to different practices) to 1 (all visits to the same practice)	
Pre/post attachment periods	12-month periods relative to date of attachment (T0)	Attachment as a potential enabling determinant
	Categorical variable with 4 periods: 0) T0–2 years (731 to 366 days before attachment) 1) T0–1 year (365 to 1 day before attachment) 2) T0 + 1 year (Attachment date to 365 days after attachment) 3) T0 + 2 years (366 to 731 days after attachment)	
Age	Age at the date of attachment, in years.	Predisposing determinant
	Categorical variable: 1–5, 6–17, 18–34, 35–54, 55–69, ≥70 years old	
Sex	Sex as indicated in patient information in billing data	Predisposing determinant
	Dichotomous variable: male, female	
Medical vulnerability	Centralized waiting lists identify a patient as medically vulnerable if they have at least one health condition among a list of 19 (e.g., diabetes, mental health problem, hypertension) or are ≥70 years old [59]. Vulnerability is assessed based on self-reported patient data upon registration, a phone evaluation by a nurse (if needed), and is verified by the family physician upon the first visit.	Need determinant
	Determined using the billing codes for attaching vulnerable/non-vulnerable patients through centralized waiting lists (non-vulnerable: 19952; vulnerable: 19951 and 19,956).	
	Dichotomous variable: vulnerable/non-vulnerable	
Charlson Comorbidity Index	Comorbidity index based on CIM-9 diagnostic codes in billing data and adjusted for age [60, 61]. Because diagnostic codes may be underreported for unattached patients facing barriers to accessing care, index was measured for each 12-month period (relative to attachment date) and the maximum value across all periods was selected for each patient.	Need determinant
	Categorical variable: low (0), medium (1–3), and high (≥4) comorbidity.	
	Categories for this study were determined upon reviewing score distribution in the included population and bivariate analyses.	
Remoteness of health region	Type of health region where patient resides. Remoteness is determined by the Ministry of Health and Social Services [62], based on population from census data, presence of large centers and influence of universities.	Enabling determinant
	Categorical variable: university (urban), peripheral, intermediary or remote	

As a measure of “realized” access to a family physician, our first dependent variable was the number of primary care visits with a family physician, per year.﻿ Our second dependent variable was the Bice-Boxerman Concentration of Care Index (COCI) [63], a widely used proxy for relational continuity of care [64]. The index measures the degree of concentration (or dispersion) of care among all providers seen by the patient. It is sensitive to shifts in distribution of visits among providers, but requires a minimum number of visits to provide reliable estimates [64]. We calculated the index for each 12-month period, relative to the attachment index date, counting visits based on primary care locations. For each 12-month period, an index was calculated only if patients had at least two primary care visits during that year, as the index is spuriously high for low users [65, 66]. If patients had fewer than two visits for a 12-month period, their index was indicated as missing for that period. We calculated an index at both family-physician and practice level. Family-physician level concentration is a common proxy for relational continuity because concentration of care is thought to be conducive to establishing a long-term relationship [64]. To identify visits to different family physicians, we used the unique physician identifier available in the RAMQ data, combined with type of delivery organization and physician speciality to identify primary care settings.

For practice-level concentration of care, we additionally used the unique identifier for the delivery organization available in the RAMQ data to identify physicians from the same practice. Practice-level concentration is relevant because team-based care in Family Medicine Groups allows patients to access other providers within their physician’s practice. This can result in high practice-level concentration despite lower physician-level concentration. Practice-level concentration may reflect having a usual source of care and may be conducive to other aspects of continuity as well (e.g. continuity of medical records, familiarity with clinic staff and team, coordination between interdisciplinary team members). RAMQ data does not, however, contain information on visits to non-physicians.

### Independent variables

The main independent variable was time pre- and post attachment. The index date for attachment (T0) was determined based on the date from the attachment billing code. Using the attachment date, T0, we established four 12-month periods: 2-years pre-attachment (T0–2 years), 1-year pre-attachment to 1 day before attachment (T0–1 year), attachment date to 1-year post attachment (T0 + 1 year); 2-years post attachment (T0 + 2 years).

Co-variables were selected based on predisposing, enabling and need determinants included in Aday and Andersen’s framework [51–53], the literature on attachment to a family physician and access to primary care as well as availability in the RAMQ database. For predisposing determinants, age at attachment date and sex were included as demographic variables. Age was categorized based on distribution and relevance to centralized waiting lists (e.g.: 70 years and over are considered medically vulnerable by centralized waiting lists). For enabling determinants, region remoteness was included because attachment and access to primary care may vary based on geography. For need determinants, analyses included whether patients were considered medically vulnerable as per the centralized waiting list criteria (see Table 1). Medical vulnerability was included because it is relevant to centralized waiting list and attachment policy in Quebec. We also included the Charlson Comorbidity Index [60, 61] to represent patients’ needs. The Charlson Comorbidity Index is originally intended to predict mortality, but has also been used to predict utilization and provides an assessment disease complexity and burden in administrative data [67, 68].

### Data analysis

We used frequencies, percentages, means and standard deviations to describe patient characteristics. We examined outcome variables graphically and with descriptive statistics. To take into account the correlation between repeated measures for a given individual in the outcome variables, we used generalized estimating equations (GEEs) which provide population-average regression coefficients [69]. GEEs also allow the inclusion of patients for whom measures are missing for certain time periods (e.g. patients attached in August 2014 who did not have a measure for T0 + 2 years). We adjusted for age, sex, region remoteness, Charlson Comorbidity Index and medical vulnerability. Robust (Huber-White sandwich) estimators were used given departure from normality and large sample size [69, 70]. For each time period, all patients with valid outcome measures were included. GEE analyses were performed in SPSS 26. Given the large sample size, statistical significance was assessed at a *p*-value < 0.01 and confidence intervals were estimated at 99% to decrease the risk of type 1 error.

For number of primary care visits, we examined the distribution graphically. Given a non-normal distribution, we estimated a negative binomial regression model with a logit link function. This type of model is well suited to estimate continuous count variables, which involve many zeros and positive integers [69]. The negative binomial model extends the Poisson model to allow for over-dispersion of data, and accounts for the extra variance [71]. In the model, we used a first order autoregressive covariance matrix. Negative binomial models estimate the logs of the expected counts, that if exponentiated, provide incidence rate ratios (IRR). IRRs represent the ratio of number of visits in one time period relative to the number of visits at T0–2 years. An IRR of 1 indicates no difference in number of primary care visits between the time periods. An IRR greater than 1 represents an increase in the number of primary care visits, whereas an IRR smaller than 1 indicates a decrease in the number of visits compared to T0–2 year.

For the Bice-Boxerman Concentration of Care Index models, we initially attempted to model the continuous form of the index, which ranges between 0 (total dispersion) and 1 (total concentration), because incremental changes have been shown to be clinically meaningful [11]. However, large proportions of patients had a score of 1, and descriptive statistics and boxplots showed a shift toward total concentration in the post-attachment period. Linear regression GEE models were unable to converge. Consequently, we estimated binomial models with logit link functions. Binomial models are appropriate to model proportion data bounded between 0 and 1 [72]. In the regression models, we used the number of primary care visits per year as the number of trials and employed unstructured covariance matrices. Exponentiated coefficients of the regression models provide odds ratios (OR). The ORs can be interpreted as the likelihood of totally concentrated care (COCI = 1.00), versus totally dispersed care during the 12-month period, compared to T0–2 years. An OR greater than 1 indicates a greater likelihood of totally concentrated care, compared to T0–2 years.

## Results

First, we present patient characteristics and selection flow chart. Second, we present the results for number of primary care visits. Third, we show results for Concentration of Care Index at the physician and practice level. For ease of interpretation, the adjusted regression models only present estimates by time period. The fully adjusted models with estimates for all covariates can be found in Additional file 1.

### Patient characteristics

**Fig. 1:**
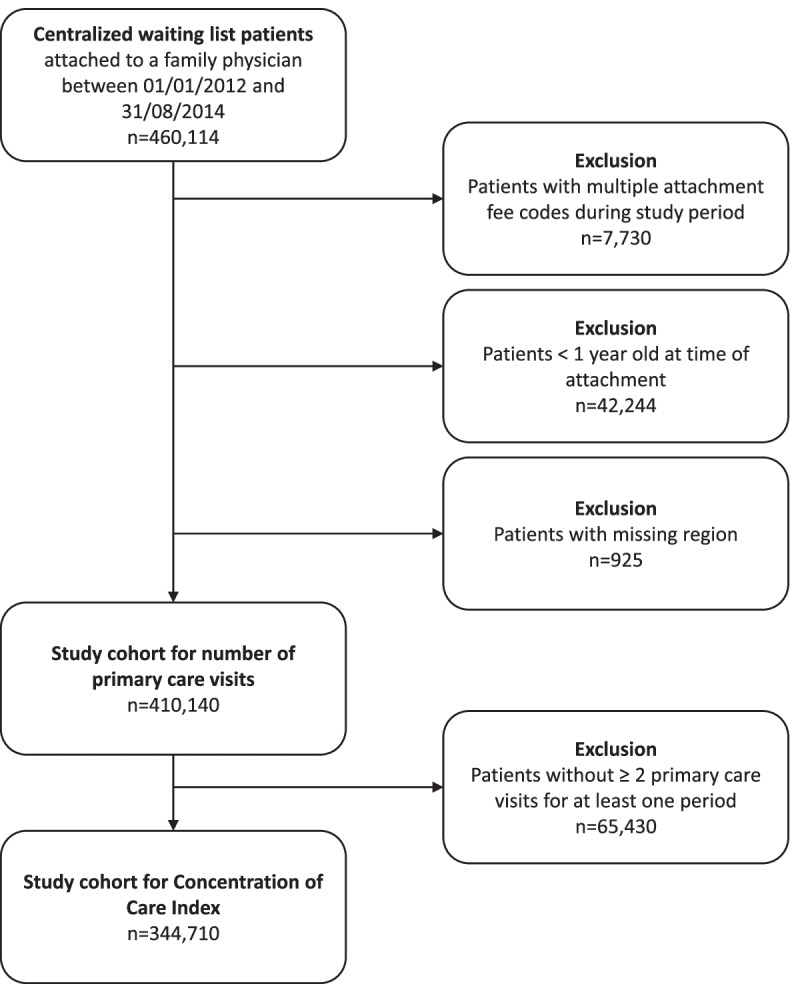
Patient selection flow chart

The study cohort for number of primary care visits was composed of 410,140 patients attached to a family physician through Quebec’s centralized waiting lists between 2012 and 2014 (see Fig. 1 for selection flow chart). The cohort for Concentration of Care Index at the physician and practice levels was composed of 344,710 patients with two or more primary care visits during at least one time period.

Table 2 presents characteristics of patients in the cohorts. For number of primary care visits, the average age of patients was 44.7 years (s.d. 22.7), 52.1% were female, 56.9% were non-vulnerable, 63.9% had a low comorbidity index, and 35.2% resided in a region with a university centre. Data on region remoteness was missing for 0.2% of patients, which were excluded from the multivariate regression models.

**Table 2:** Characteristics of included centralized waiting list patients, attached to a family physician between 2012 and 2014

Variables	Cohort for number of primary care visits (*n* = 411,065) n (%)	Cohort for Concentration of Care Index (*n* = 345,502) n (%)
**Age**		
1–5	20,602 (5.0%)	16,696 (4.8%)
6–17	39,614 (9.6%)	26,773 (7.7%)
18–34	82,307 (20.0%)	65,793 (19.0%)
35–54	117,003 (28.5%)	97,506 (28.2%)
55–69	89,414 (21.8%)	79,553 (23.0%)
70+	62,125 (15.1%)	59,181 (17.1%)
**Sex**		
Male	196,739 (47.9%)	158,963 (46.0%)
Female	214,326 (52.1%)	186,539 (54.0%)
**Medical vulnerability**		
Non-vulnerable	233,965 (56.9%)	181,330 (52.5%)
Vulnerable	177,100 (43.1%)	164,172 (47.5%)
**Charlson Comorbidity Index**		
Low (0)	262,537 (63.9%)	207,265 (60.0%)
Medium [1–3]	123,021 (29.9%)	114,278 (33.1%)
High (4+)	25,507 (6.2%)	23,959 (6.9%)
**Region remoteness**		
Remote	63,256 (15.4%)	48,467 (14.0%)
Intermediary	100,009 (24.3%)	83,747 (24.2%)
Peripheral	102,254 (24.9%)	87,146 (25.2%)
University	144,621 (35.2%)	125,350 (36.3%)
Missing	925 (0.2%)	792 (0.2%)

For concentration of care, the average age was 46.5 years (s.d. 22.7), 64.0% were female, 52.5% were non-vulnerable, 60.0% had a low comorbidity index and 36.3% resided in a region with a university center. Data on region remoteness was missing for 0.2% of patients. Because we excluded low users for concentration of care analyses (without two or more primary care visits during at least one period), more patients were vulnerable (47.5% vs. 43.1%), more patients had medium comorbidity (33.1% vs. 29.9%), a slightly larger proportion were female (54.0% vs. 52.1%) and they were slightly older (46.5 vs. 44.7 years).

Patients excluded from the Concentration of Care Index analyses (low users without two or more primary care visits during at least one period) were younger (34.2 years old on average (s.d. 19.8)). A larger proportion of excluded low users were male (58.4%), non-vulnerable (82.6%), low comorbidity (85.1%) and from remote areas (20.7%).

### Number of primary care visits

Table 3 presents descriptive statistics as well as results from the unadjusted and adjusted negative binomial regression models for the number of primary care visits per year. The descriptive statistics indicate minimal change in the years before attachment (1.77 and 1.76 visits). In the first year after attachment, we observed a substantially higher average number of primary care visits (3.43 visits). The average increase between T0–2 years and T0 + 1 year was 1.66 visits, which represents more than just the first intake visit needed to formalize attachment. In the second year after attachment, the average number of primary care visits remains substantially higher (2.20 visits) than before attachment, but slightly lower than in the first year after attachment.

**Table 3:** Results for number of primary care visits by time period: descriptive statistics and GEE repeated measures model estimates, unadjusted and adjusted for potentially predisposing, need and enabling covariates

	Pre-attachment		Post-attachment	
	T0–2 years	T0–1 year	T0 + 1 year	T0 + 2 years
n^a^	406,001	410,140	410,140	320,428
*Number of primary care visits*				
Mean (s.d.)	1.77 (2.68)	1.76 (2.60)	3.43 (3.08)	2.20 (2.70)
99% CI	1.76–1.78	1.75–1.77	3.42–3.44	2.19–2.21
*Unadjusted model*				
Exp (β) (IRR)	1.00 (ref.)	0.99	1.94	1.24
99% CI		0.99–1.00	1.93–1.95	1.24–1.25
*p*-value		< 0.001	< 0.001	< 0.001
*Adjusted model*^*b*^				
Exp (β) (IRR)	1.00 (ref.).	1.01	2.03	1.29
99% CI		1.00–1.01	2.02–2.04	1.28–1.30
*p*-value		< 0.001	< 0.001	< 0.001

^a^Number of patients with valid data in the 12-month time period

^b^Adjusted for age, sex, Charlson Comorbidity Index, medical vulnerability and region remoteness

In our unadjusted model, before attachment, we observed a very small 1% decrease in the number of primary care visits (IRR = 0.99, *p* < 0.001). Following attachment, the number of visits increased by 94% (IRR = 1.94, *p* < 0.001) in the first year, and by 24% (IRR = 1.24, *p* < 0.001) in the second year after attachment, compared to T0–2 years. After adjusting for age, sex, medical vulnerability, Charlson Comorbidity Index and region remoteness, the results remain similar: we observed a minimal change in the year before attachment (IRR = 1.01, *p* < 0.001), a two-fold increase (IRR = 2.03, *p* < 0.001) in the first year after attachment, and a 29% increase in the second year after attachment (IRR = 1.29, *p* < 0.001), compared to 2 years before attachment.

### Bice-Boxerman concentration of care index

Table 4 presents descriptive statistics and results from the unadjusted and adjusted binomial regression models for the Concentration of Care Index (COCI) at the family physician level. It should be noted that we only calculated the Bice-Boxerman Concentration of Care Index when a patient had at least two primary care visits for a given time period. Due to the post-attachment increase in number of primary care visits presented above, the number of patients with valid COCI measures (≥2 more primary care visits per period) varies across periods.

**Table 4:** Results for the Bice-Boxerman Concentration of Care Index at the physician level by time period: descriptive statistics and GEE repeated measures model estimates, unadjusted and adjusted for potentially predisposing, need and enabling covariates

	Pre-attachment		Post-attachment	
	T0–2 years	T0–1 year	T0 + 1 year	T0 + 2 years
Number of patients with at least 2 primary care visits^a^	155,086	156,760	299,594	155,729
*Concentration of Care Index – Physician-level*				
Mean (s.d.)	0.56 (0.43)	0.45 (0.42)	0.72 (0.37)	0.64 (0.41)
99% CI	0.55–0.56	0.44–0.45	0.71–0.72	0.63–0.64
Proportion (%) of patients with totally concentrated primary care (COCI = 1)	44	32	59	51
Proportion (%) of patients with totally dispersed primary care (COCI = 0.00)	24	31	10	17
*Number of primary care visits*^*b*^				
Mean (s.d.)	2.06 (2.82)	2.05 (2.74)	3.92 (3.12)	2.53 (2.80)
99% CI	2.05–2.07	2.04–2.06	3.90–3.93	2.52–2.54
*Unadjusted regression*				
Exp (β)	1.00 (ref.)	0.74	1.46	1.18
99% CI		0.71–0.76	1.38–1.53	1.13–1.23
*p*-value		< 0.001	< 0.001	< 0.001
*Adjusted regression*^*c*^				
Exp (β)	1.00 (ref.)	0.76	1.53	1.22
99% CI		0.74–0.79	1.47–1.60	1.16–1.28
*p*-value		< 0.001	< 0.001	< 0.001

^a^Number of patients with valid data in the 12-month time period

^b^Number of primary care visits is not included in this analysis, but ≥2 visits were required for a valid index. Number of visits is provided to indicate primary care utilization per time period

^c^Adjusted for age, sex, Charlson Comorbidity Index, medical vulnerability and region remoteness

Before attachment, only 38% of the cohort had a minimum of two primary visits (T0–2 years: *n* = 155,086/406,101; T0–1 year: *n* = 156,760/410,140). Amongst those with at least two visits, the mean Concentration of Care Index at T0–2 years was 0.56, with 44% of patients having all care concentrated with a single physician (i.e., COCI = 1.00), and 24% having completely dispersed care (i.e., COCI = 0.00). One year before attachment, the mean COCI was 0.45, only 32% of patients had all their care concentrated with a single physician, 31% had all their care dispersed between different physicians, and the likelihood of total concentration decreased significantly (unadjusted OR = 0.74, *p* < 0.001).

In contrast, in the first post-attachment year, 73% of our cohort had at least two primary care visits (*n* = 299,594/410,140), the average COCI increased to 0.72, 59% of patients had all their care concentrated with a single physician and only 10% had totally dispersed care. During the first post-attachment year, we found a clinically and statistically significant 46% increase (unadjusted OR = 1.46) in the odds of having all primary care visits concentrated with a single physician, as opposed to having dispersed care, compared to the reference year before attachment. In second year after attachment, 49% of patients had at least two primary care visits (*n* = 155,729/320,428). A higher likelihood of concentrated care was maintained in the second year after attachment, though with a more modest 18% increase compared to T0–2 years (unadjusted OR = 1.18). The effects were slightly stronger after adjusting for potential predisposing, need and enabling covariates: the likelihood of concentrated care increased by 53% and 22% in the first and second year after attachment respectively, compared to T0–2 years.

Results for the Concentration of Care Index at practice level are presented in Table 5. Before attachment, the average COCI were 0.78 (T0–2 years) and 0.72 (T0–1 year), and 67% (T0–2 years) and 59% (T0–1 year) of patients had all their primary concentrated with a single practice. A larger proportion of patients had care totally concentrated with a single practice (67%, 59%) than a single physician (44%, 32%). The adjusted likelihood of perfect a Concentration of Care Index decreased by 20% (OR = 0.80, *p* < 0.001) in the year before attachment, compared to T0–2 years.

**Table 5:** Results for the Bice-Boxerman Concentration of Care Index at the practice level by time period: descriptive statistics and GEE repeated measures model estimates, unadjusted and adjusted for potentially predisposing, need and enabling covariates

	Pre-attachment		Post-attachment	
	T0–2 years	T0–1 year	T0 + 1 year	T0 + 2 years
Number of patients with at least 2 primary care visits^a^	155,086	156,760	299,594	155,729
*Concentration of care index – Practice-level*				
Mean (s.d.)	0.78 (0.34)	0.72 (0.37)	0.84 (0.30)	0.82 (0.32)
99% CI	0.78–0.79	0.71–0.72	0.83–0.84	0.81–0.82
Proportion (%) of patients with totally concentrated primary care (COCI = 1)	67	59	73	72
Proportion (%) of patients with totally dispersed primary care (COCI = 0.00)	9	12	5	7
*Number of primary care visits*^*b*^				
Mean (s.d.)	2.06 (2.82)	2.05 (2.74)	3.92 (3.12)	2.53 (2.80)
99% CI	2.05–2.07	2.04–2.06	3.90–3.93	2.52–2.54
*Unadjusted regression*				
Exp (β)	1.00 (ref.)	0.78	1.17	1.12
99% CI		0.75–0.81	1.11–1.22	1.06–1.18
*p*-value		< 0.001	< 0.001	< 0.001
*Adjusted regression*^*c*^				
Exp (β)	1.00 (ref.)	0.80	1.19	1.15
99% CI		0.77–0.83	1.14–1.25	1.09–1.22
*p*-value		< 0.001	< 0.001	< 0.001

^a^Number of patients with valid data in the 12-mont time period

^b^Number of primary care visits is not included in this analysis, but ≥2 visits were required for a valid index. Number of visits is provided to indicate primary care utilization per time period

^c^Adjusted for age, sex, Charlson Comorbidity Index, medical vulnerability and region remoteness

After attachment, the average COCI was 0.84 (T0 + 1 year) and 0.82 (T0 + 2 years), with 73% and 72% of patients having all their care concentrated with one practice. Adjusting for age, sex, Charlson Comorbidity Index, medical vulnerability and region remoteness in our binomial regression model did not substantially change the estimated coefficients. After attachment, our results show a 19% increase in the odds of having all primary care visits concentrated with one practice versus all visits being dispersed (OR = 1.19, *p* < 0.001) in the first year and a 15% increase (OR = 1.15, *p* < 0.001) in the second year, compared to 2 years before attachment. These increases are more modest than at the physician level.

## Discussion

This study evaluated changes in access to and continuity of primary care associated with attachment to a family physician, through Quebec’s centralized waiting lists for unattached patients. In accordance with our hypothesis and policy objectives, results show an increase in primary care utilization and in concentration of care at the physician and practice level in the 2 years following attachment. This suggests an improvement in access to and continuity of primary care after attachment. To the best of our knowledge, this study is the first to evaluate changes in access associated with attachment, comparing patients before and after attachment to a family physician.

### Access to primary care

With regard to access, results show a statistically significant and substantial increase in the number of primary care visits to a family physician. Compared to the reference year, the number of primary care visits doubled in the first year after attachment, and was 29% higher in the second year. The large increase in the post-attachment year is partly attributable to the requirement that all patients have a first intake visit with their new family physician to confirm attachment. This intake visit is often longer, allowing patients to share their medical history, fill out administrative forms and receive preventive care. However, the fact that, on average, visits increased by more than one and that the increase was sustained in the second year following attachment suggests a real improvement in access to primary care.

This increase in primary care utilization suggests an improvement in “realized” access, or successful entry into primary care, for previously unattached patients. Patients without a regular provider often avoid health care, have unmet needs, face difficulties accessing preventive care, and accumulate health problems [2, 7, 18–20, 73]. The increase in the number of primary care visits after attachment may also reflect a backlog of unmet needs and visits to follow up on preventive care (e.g., appointment to discuss blood test results). Formal attachment to a family physician, through centralized waiting lists, seems to act as an enabling determinant of “realized” access to primary care, as conceptualized in Aday and Andersen’s framework for the study of access [53].

### Continuity of care

As measured by the Bice-Boxerman Concentration of Care Index, our results show a post-attachment increase in the odds of having all primary care visits concentrated versus all visits being dispersed. The increase was observed at both the physician and practice level. These results suggest that continuity of care improves following attachment. This finding contrasts with results of a recent study conducted in Ontario, Canada, where transitioning from a traditional fee-for-service model to an enhanced fee-for-service model featuring patient attachment was associated with a small decrease in continuity of care at the physician level [37]. However, the decrease observed in Ontario may be due to the transition to a team-based structure in which physicians are encouraged to see their colleagues’ attached patients [37]. The results reported in their study do not provide a comparable assessment changes associated with attachment, because it is likely that patients had a regular primary care provider before the implementation of the new model.

We observed a more marked increase in the Concentration of Care Index at family physician level than at practice level. This may be due to unattached patients having a regular source of care, such as a walk-in clinic, despite not having a regular family physician [2, 73]. It also suggests a post-attachment improvement in the concentration of care with a single provider, which may support better relational continuity [54, 74]. While continuity is desirable at both physician and practice level, previous studies have found that having a regular primary care provider, as opposed to only having a usual source of care or regular practice, is associated with more preventive care [75, 76], better chronic disease and medication management [77], and improved access to care [3]. Previous work suggests that relational continuity may be more difficult to improve than information or management continuity [11] and that having a named primary care provider may be conducive to the development of a patient-provider relationship [74]. Although we cannot make inferences about the patient-provider relationship based on our study, our findings suggest that attachment improves concentration of care at the family physician level, which is conducive to relational continuity.

### Potential mechanisms of attachment

The results of this study suggest that attachment through centralized waiting lists improves access to and continuity of primary care. While attachment remains understudied, there are several potential underlying mechanisms for these effects. Attachment may improve patients’ ability to navigate health care services by clearly identifying their family physician and practice. Attachment also allows patients to access appointments reserved for patients attached to family physicians in a practice. Family physicians and practices in Quebec are incentivized to attach patients and to provide these patients with most of their care; as a result, many physicians reserve their appointments for attached patients. Attachment may also make family physicians feel more accountable for assuring access to care for their patients, enable physicians and practices to gain a better understanding of their patient panel and balance supply and demand. In Family Medicine Groups, the total number of patients attached to Group physicians is linked to additional resources for allied health professionals and extended after-hours coverage, which may improve access to and continuity of care. Moreover, the formalization of attachment in a signed agreement may be the first step in building a strong, long term patient-provider relationship [35, 78–81] that is central to continuity of care. This confirmation of the patient-provider relationship is perhaps even more important for patients attached through centralized waiting lists as the relationship is formally arranged between patients and physicians who have never met, rather than developed organically through multiple encounters over time.

### Implications for policy

In terms of policy, attachment has been implemented across Quebec, along with centralized waiting lists to help unattached patients become attached to a family physician. The province aims to have 85% of the population attached to a family physician with the explicit intent to improve access to and continuity of primary care [82, 83]. Every year, over $23 million is paid to family physicians in premiums for attachment [84] and more than 1.3 million patients have been attached through centralized waiting lists since their implementation [85]. Six other provinces across Canada have implemented similar centralized waiting lists [43] and they also feature in various forms in other countries. Evaluating changes associated with attachment through centralized waiting lists is therefore relevant to inform health care policy.

Our results suggest that centralized waiting lists improve access to and continuity of primary care for patients who are attached to a family physician. However, the large proportion of relatively healthy patients in our cohorts (52.5 and 56.9% non-vulnerable patients; 60.0 and 63.9% low comorbidity) also indicates that challenges remain to attach patients with medical conditions and for whom attachment may have the most benefits. This is coherent with prior research that has found evidence that family physicians “cherry-pick” healthier patients for attachment, avoiding patients with more complex health needs (e.g. mental health problems, intellectual disability) [44, 46–49].

Attachment, which is considered a key feature of the patient medical home [86–88] and a building block of high performing care [31], has been implemented in countries such as Canada, Denmark, France, Germany, Ireland, Israel, Italy, the Netherlands, Norway, Sweden, Switzerland, New Zealand, the United Kingdom, Mongolia, Costa Rica, and the United States [30, 33, 34, 36]. Other research conducted in Quebec suggests that, at the populational level, attachment policies from 2003 and 2009 have not meaningfully impacted measures of having a regular physician and concentration of care [42]. It is possible that during early implementation, attachment policies mainly formalized pre-existing informal relationships between providers and patients, and may not have benefited patients without a regular provider or usual source of care [42, 44]. Evaluating attachment through centralized waiting lists is a valuable contribution to previous evidence: it allows us to observe changes in primary care utilization and concentration of care specifically amongst previously unattached patients who become attached. These patients were unlikely to have had a regular family physician before attachment: registering on the centralized waiting list suggests that they were actively seeking a primary care provider. Our findings provide novel insight into the changes associated with attachment in patients previously without a regular primary care provider. This indicates the potential of attachment policies, once they reach patients without a regular provider. Reaching these patients, through better implementation of attachment policies and other primary care policies, is an important policy priority given their poor access to and continuity of primary care [2, 7, 18–21]. The voluntary nature of attachment policies and “cherry-picking” of healthier patients may limit their populational impact [42, 49]. Future studies should explore how attachment policies may be improved to maximize their impact.

Our findings offer insight into how many primary care visits newly attached patients may generate, which can be useful for planning primary care delivery at the provider, practice and system level. We find that attachment through centralized waiting lists may generate approximately 166,000 additional primary care visits per 100,000 population in the first year following attachment, and about 43,100 additional primary care visits in the second year following attachment. This increase in primary care visits may in turn help reduce avoidable emergency department visits, as emergency departments are sometimes used as substitutes when patients are unable to access primary care [26, 89–91]. Future research should examine to extent to which attachment helps reduce emergency department visits.

Although we find that primary care is more concentrated after attachment, there remains some care dispersion. This may be due to lack of timely access to primary care even when patients have a regular family physician, and to a preference for timely access for minor health needs over seeing their regular provider [24, 92, 93]. Post attachment concentration of care may be further improved through continuity of care targets recently implemented in Quebec [82, 83], or organizational interventions enabling more timely access to family physicians. Advanced access is increasingly widespread in Quebec and has shown promising results to improve access to and continuity with family physicians and other primary care providers [82]. It is an organizational model that promotes timely access to care through balancing supply and demand for care, adjusting available appointments and assessing urgency of patients’ needs, reviewing appointment systems, integrating interprofessional collaboration and developing contingency plans [82]. Further support to implement advanced access may help achieve the full benefits of attachment, by helping improve timely access  to attached family physicians, limiting patients’ need to seek care elsewhere.

Finally, our results highlight that unattached patients have fewer primary care visits and more dispersed care, which suggests more unmet needs, lack of access and poorer continuity of primary care. Creating more options for unattached patients to access care, particularly while they are waiting for attachment on the centralized waiting list, is essential to meet their primary care needs.

### Strengths and limitations

This study has several notable strengths. By using administrative billing data from the provincial health insurance board, the study included a populational cohort of nearly all patients attached to family physicians through centralized waiting lists between 2012 and 2014. This included patients from across different regions and age groups; many other studies on access to a family physician are restricted to specific populations. In addition, our longitudinal design allowed us to observe pre-post attachment changes in our outcomes, reducing to some extent the risk of bias related to unmeasured differences between attached and unattached patients. Furthermore, RAMQ billing data captures most medical visits to family physicians in Quebec, providing good internal validity for the outcome variables used (concentration of care and number of primary care visits). Moreover, medical vulnerability (presence of at least one health condition or being 70 or older) was determined based on fee code for attachment, which is assessed by both the centralized waiting list staff and family physician, conferring good validity to the measure. Patients’ health needs were also measured through the Charlson Comorbidity Index: a validated measure for use in administrative billing data that is widely used.

The study also has a number of limitations. First, because we used administrative billing data, we were limited in the availability of patient-level characteristics included as covariates in our analyses. We were therefore unable to include characteristics such as immigration status, ethnicity, or socioeconomic status which may influence the relationship between attachment and access to and continuity of primary care. The covariates for which we did adjust our models did not however substantially change the estimates.

Second, over half of our cohorts (52.5 and 56.9%) were patients deemed non-vulnerable (no medical conditions). This large proportion of healthy patients attached through the centralized waiting lists is slightly lower than reported in a previous study (about 70% non-vulnerable patients attached through centralized waiting lists) [44]. This difference is likely due to the time period included in the previous study during which centralized waiting lists policies were more conducive to cherry-picking by family physicians [44]. Other studies conducted during our study period have similar proportions of non-vulnerable attached patients (54–60%) [45, 47]. This large proportion of non-vulnerable patients may limit the generalizability of our results. However, we believe this sample appropriately reflects the general population which is targeted by attachment and centralized waiting lists policies. The largely healthy sample also suggests that our estimates for primary care utilization and concentration of care are conservative and that observed changes are more likely due to attachment than to changes in patients’ health needs.

Third, there are limitations associated with using the Bice-Boxerman Concentration of Care Index. The index is spuriously high for low users (< 2 visits per year). Hence, we excluded low users from our analyses for Concentration of Care Indexes. We reported the characteristics of patients included for the concentration of care analyses in Table 2 and described excluded low users. Low users were younger and a larger proportion was male, non-vulnerable, low comorbidity and from remote areas, compared to patients included in the analyses. Excluding them may have introduced selection bias in our results. Younger patients, male patients, patients from rural areas and patients with fewer chronic diseases have been shown to have lower relational continuity [94, 95]. Future work should further explore how attachment influences continuity for these patients using measures appropriate for low users. It should also be noted that in the first post-attachment year, the number of patients with at least two visits increased substantially as the average number of primary care visits increased, which may have influenced results. To show this variation, we reported the percentage of patients with at least two visits and average number of primary care visits for each time period alongside the Concentration of Care results. The Bice-Boxerman Concentration of Care Index also has limitations as a measure for continuity of care: while concentration is conducive to relational continuity it remains a proxy. It is possible that more concentrated care could be due to difficulty accessing primary care elsewhere or convenience of seeing their attached family physician rather than to relational continuity.

Fourth, our data was limited to medical services delivered by fee-for-service physician and, therefore, does not include primary care delivered by salaried physicians, the private sector or allied health providers. This limits the generalizability our results beyond fee-for-service physicians.

Fifth, our main outcomes were number of primary care visits (“realized” access) and Concentration of Care Index at the physician and practice levels (continuity of care), commonly used measures of access and continuity plausibly influenced by attachment. It would be relevant for future studies to evaluate effects of attachment on other measures such as emergency department visits and patient-reported experience and outcomes measures.

Sixth, due to our observational study design and lack of control group, there is a risk of historical bias, as changes in the health system happening during our study period may have influenced our results. However, our results show substantial initial improvements in study outcomes in the year following attachment, which are sustained at slightly lower levels in the second year after attachment. This pattern may also be consistent with an episode of illness, which could have accelerated attachment through the centralized waiting lists’ prioritization process and would explain increase in utilization [49, 96]. However, we observe this pattern for non-vulnerable patients as well who are considered generally healthy. Therefore, we are fairly confident that the findings presented in this paper are associated with attachment.

Finally, the follow-up period is only 2 years. Findings should be interpreted with caution and cannot be generalized beyond this period. Nevertheless, our longitudinal study design provides a unique contribution to the literature on access to a family physician. Future research should examine whether these results are sustained beyond 2 years.

## Conclusion

This study is the first to examine changes in access to and continuity of primary care associated with attachment through centralized waiting lists for unattached. Results show an increase in the number of primary care visits and the concentration of care (at family physician and practice level) after patient attachment to a family physician. Findings support attachment as an important building block of primary care.

## Supplementary Information

**Additional file 1.** Fully adjusted regression models.

## Data Availability

The dataset analysed during the current study is not publicly available, as per the agreement with the *Commission d’accès à l’information*, which regulates access to administrative billing data in the province of Quebec. The corresponding author, Mylaine Breton, should be contacted for data enquiries.

## Abbreviations

CI: Confidence interval
COCI: Concentration of care index
FPL: Family physician-level
GEE: Generalized estimating equations
IRR: Incidence rate ratio
OR: Odds ratio
PL: Practice-level
RAMQ: Régie de l’assurance maladie du Québec
S.d.: Standard deviation
STROBE: Strengthening the Reporting of Observational Studies in Epidemiology
