# Supplementary material for: Improved access to and continuity of primary care after attachment to a family physician: longitudinal cohort study on centralized waiting lists for unattached patients in Quebec, Canada
Source: BMC Prim Care. 2022 Sep 16;23:238. doi: 10.1186/s12875-022-01850-4 (PMC9482231; doi:10.1186/s12875-022-01850-4)
Supplement: Supplementary file 1 — Additional file 1. Fully adjusted regression models. [file 12875_2022_1850_MOESM1_ESM.docx]

Additional file 1 – Fully adjusted regression models

| **Variables** | **Number of primary care visits^a^** | | | **Concentration of care index – Physician level^b^** | | | **Concentration of care index – Practice level^c^** | | |
| --- | --- | --- | --- | --- | --- | --- | --- | --- | --- |
|  | Exp (B) | 99% CI | p-value | Exp (B) | 99% CI | p-value | Exp (B) | 99% CI | p-value |
| **Constant** | 1.275 | 1.253-1.298 | <0.001 | 0.397 | 0.372-0.424 | <0.001 | 2.258 | 2.110-2.416 | <0.001 |
| **Time** |  |  |  |  |  |  |  |  |  |
| Pre-attachment T0-2 years | Ref. | Ref. | Ref. | Ref. | Ref. | Ref. | Ref. | Ref. | Ref. |
| Pre-attachment  T0-1 year | 1.008 | 1.002-1.015 | <0.001 | 0.762 | 0.735-0.789 | <0.001 | 0.802 | 0.772-0.833 | <0.001 |
| Post-attachment T0+1 year | 2.029 | 2.016-2.041 | <0.001 | 1.534 | 1.474-1.597 | <0.001 | 1.192 | 1.140-1.246 | <0.001 |
| Post-attachment T0+2 years | 1.293 | 1.284-1.302 | <0.001 | 1.219 | 1.164-1.277 | <0.001 | 1.151 | 1.090-1.215 | <0.001 |
| **Age** |  |  |  |  |  |  |  |  |  |
| 1-5 | Ref. | Ref. | Ref. | Ref. | Ref. | Ref. | Ref. | Ref. | Ref. |
| 6-17 | 0.597 | 0.586-0.609 | <0.001 | 1.457 | 1.002-2.118 | 0.10 | 1.473 | 1.185-1.832 | <0.001 |
| 18-34 | 0.934 | 0.917-0.950 | <0.001 | 2.195 | 2.078-2.319 | <0.001 | 1.329 | 1.258-1.404 | <0.001 |
| 35-54 | 0.922 | 0.907-0.938 | <0.001 | 2.775 | 2.613-2.946 | <0.001 | 1.502 | 1.415-1.594 | <0.001 |
| 55-69 | 0.950 | 0.933-0.966 | <0.001 | 3.533 | 3.297-3.786 | <0.001 | 1.938 | 1.814-2.070 | <0.001 |
| 70+ | 1.120 | 1.099-1.142 | <0.001 | 6.063 | 5.437-6.762 | <0.001 | 2.819 | 2.603-3.053 | <0.001 |
| **Sex** |  |  |  |  |  |  |  |  |  |
| Male | Ref. | Ref. | Ref. | Ref. | Ref. | Ref. | Ref. | Ref. | Ref. |
| Female | 1.316 | 1.307-1.326 | <0.001 | 1.003 | 0.949-1.060 | 0.896 | 0.956 | 0.912-1.002 | 0.013 |
| **Medical vulnerability** |  |  |  |  |  |  |  |  |  |
| Non-vulnerable | Ref. | Ref. | Ref. | Ref. | Ref. | Ref. | Ref. | Ref. | Ref. |
| Vulnerable | 1.344 | 1.332-1.356 | <0.001 | 1.304 | 1.215 | <0.001 | 1.098 | 1.053-1.146 | <0.001 |
| **Charlson Co-morbidity index** |  |  |  |  |  |  |  |  |  |
| Low (0) | Ref. | Ref. | Ref. | Ref. | Ref. | Ref. | Ref. | Ref. | Ref. |
| Medium (1-3) | 1.344 | 1.332-1.356 | <0.001 | 0.875 | 0.840-0.912 | <0.001 | 0.908 | 0.870-0.947 | <0.001 |
| High (4+) | 1.326 | 1.305-1.346 | <0.001 | 1.030 | 0.894-1.186 | 0.594 | 1.077 | 0.966-1.199 | 0.078 |
| **Region remoteness** |  |  |  |  |  |  |  |  |  |
| University | Ref. | Ref. | Ref. | Ref. | Ref. | Ref. | Ref. | Ref. | Ref. |
| Peripheral | 0.991 | 0.983-1.000 | 0.013 | 0.975 | 0.925-1.028 | 0.223 | 1.093 | 1.041-1.147 | <0.001 |
| Intermediary | 0.929 | 0.920-0.937 | <0.001 | 1.160 | 1.082-1.242 | <0.001 | 1.334 | 1.264-1.408 | <0.001 |
| Remote | 0.794 | 0.785-0.803 | <0.001 | 1.231 | 1.109-1.367 | <0.001 | 1.748 | 1.595-1.914 | <0.001 |
| 1. Generalized estimating equation, negative binomial regression with logit link function, first order autoregressive (AR1) covariance matrix, robust estimators. 2. Generalized estimating equation, binomial regression with logit link function, unstructured covariance matrix, robust estimators. 3. Generalized estimating equation, binomial regression with logit link function, unstructured covariance matrix, robust estimators. | | | | | | | | | |
